# Materials design strategies for green thermoelectric generators enabling sustainable and scalable energy harvesting

**DOI:** 10.1016/j.isci.2026.117705

**Published:** 2026-10-01

**Authors:** Nuur Syahidah Sabran, Iman Aris Fadzallah, Nguyen Van Toan, Takahito Ono, Mohd Faizul Mohd Sabri, H.K. Jun

**Affiliations:** 1 Department of Electrical and Electronic Engineering, Faculty of Engineering, Technology and Built Environment (FETBE), UCSI University, Kuala Lumpur, Malaysia; 2 Centre for Sustainable Mobility Technologies, Universiti Tunku Abdul Rahman, Sungai Long Campus, Bandar Sungai Long, Kajang 43000, Malaysia; 3 Graduate School of Engineering, Tohoku University, AzaAoba 6-6-01, Aramaki, Aoba-ku, Sendai 980-8579, Japan; 4 Faculty of Mechanical Engineering, Industrial University of Ho Chi Minh City, Ho Chi Minh City, Vietnam; 5 Department of Mechanical Engineering, Faculty of Engineering, Universiti Malaya, Kuala Lumpur 50603, Malaysia; 6 Centre for Advanced and Sustainable Materials Research, Universiti Tunku Abdul Rahman, Sungai Long Campus, Bandar Sungai Long, Kajang 43000, Malaysia; 7 Department of Mechanical and Materials Engineering, Lee Kong Chian Faculty of Engineering and Science, Universiti Tunku Abdul Rahman, Sungai Long Campus, Jalan Sungai Long, Bandar Sungai Long, Kajang 43000, Malaysia

**Keywords:** green materials, thermoelectric generators, sustainable materials design, green synthesis, biomass-derived materials

## Abstract

Thermoelectric generators (TEGs) harvest low-grade heat without moving parts and can be integrated into compact, flexible, or distributed systems. Progress in this field is typically measured through figure of merit, *ZT*, and the power factor, yet materials with high *ZT* often depend on scarce elements, toxic precursors, high-temperature processing, or difficult end-of-life recovery-considerations that matter for technologies intended to support sustainable energy systems. This perspective examines green thermoelectric design as a coupled materials, process, device, and life cycle problem, considering how aqueous processing, bio-assisted synthesis, hydrothermal methods, electrochemical deposition, and printable processing shape microstructure, defect chemistry, and charge-heat transport in conductive polymers, carbon nanotube hybrids, biomass-derived carbons, recycled inorganic compounds, and ionic thermoelectric systems. We propose a design framework that combines material abundance, process energy, solvent and waste burden, device lifetime, manufacturability, and circularity, and identify where environmentally responsible TEGs can deliver useful power with lower total burden.

## Graphical abstract

**Figure:**
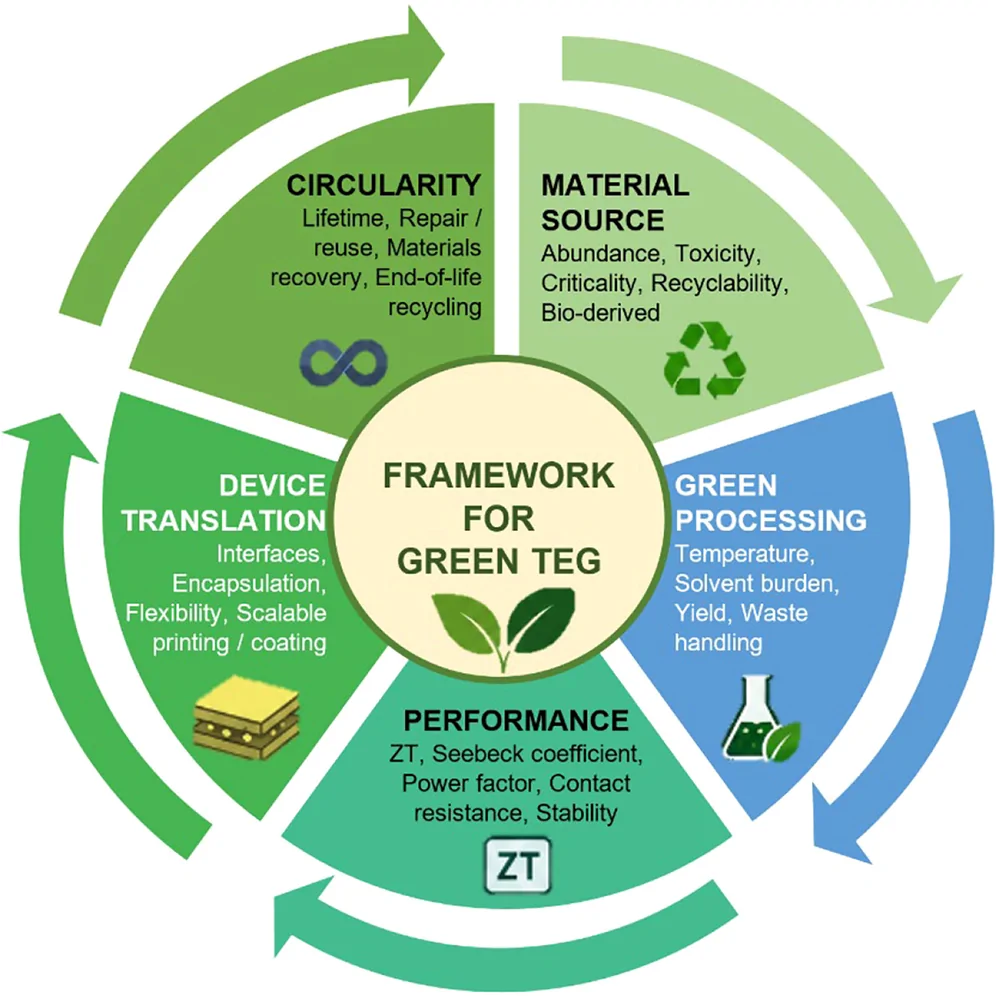

## Teaser

Applied sciences; Energy engineering; Energy systems

## Introduction

Thermoelectric generators (TEGs) convert a temperature difference into electrical power via the Seebeck effect. TEGs offer several practical advantages, including solid-state operation, silent performance, compact size, and the ability to harvest energy from heat that would otherwise be wasted. Low-grade heat from buildings, industrial surfaces, vehicles, wearable systems, and distributed electronics is therefore a natural target for TEGs. The concern is that many conventional thermoelectric materials carry their own sustainability burden. Common high-performance systems like bismuth telluride (*Bi*_*2*_*Te*_*3*_), lead telluride (*PbTe*), and silicon-germanium (*SiGe*) rely on scarce or toxic elements, demanding purification routes, and energy-intensive processing. A device designed to recover waste heat can therefore lose part of its environmental and sustainability values before it is installed.^1^

This predicament has pushed the field toward green-source and green-processed thermoelectric materials. Biomass-derived carbon, conductive polymers, cellulose-based substrates, recycled inorganic compounds, and ionic thermoelectric systems all offer routes away from a high ZT framework.^2^^,^^3^^,^^4^ Unfortunately, they also introduce new challenges. Many green materials have modest electrical conductivity, unstable doping, poor humidity tolerance, low output power, or uncertain scalability. In some cases, a material is described as green because one component is renewable, while the solvent, post-treatment, filler, or end-of-life route remains environmentally questionable. The central issue is therefore not on scalability of green thermoelectrics but how they are perceived. A material with moderate output but low-temperature processing, abundant precursors, and a realistic recycling route may be more useful in some applications than a higher-*ZT* material that depends on rare elements or demanding processing. Conversely, a bio-derived source does not automatically make a thermoelectric material sustainable if high-temperature activation, hazardous solvents, or short lifetime dominates the environmental cost.

We argue that green thermoelectrics should be evaluated through a coupled performance-sustainability framework in which *ZT* is only one criterion alongside process energy, solvent and water consumption, material criticality, manufacturability, lifetime, and end-of-life recovery. Figure 1 summarizes this framework. It is intended to shift green TEG research from isolated performance claims toward accountable materials design.

**Figure 1:**
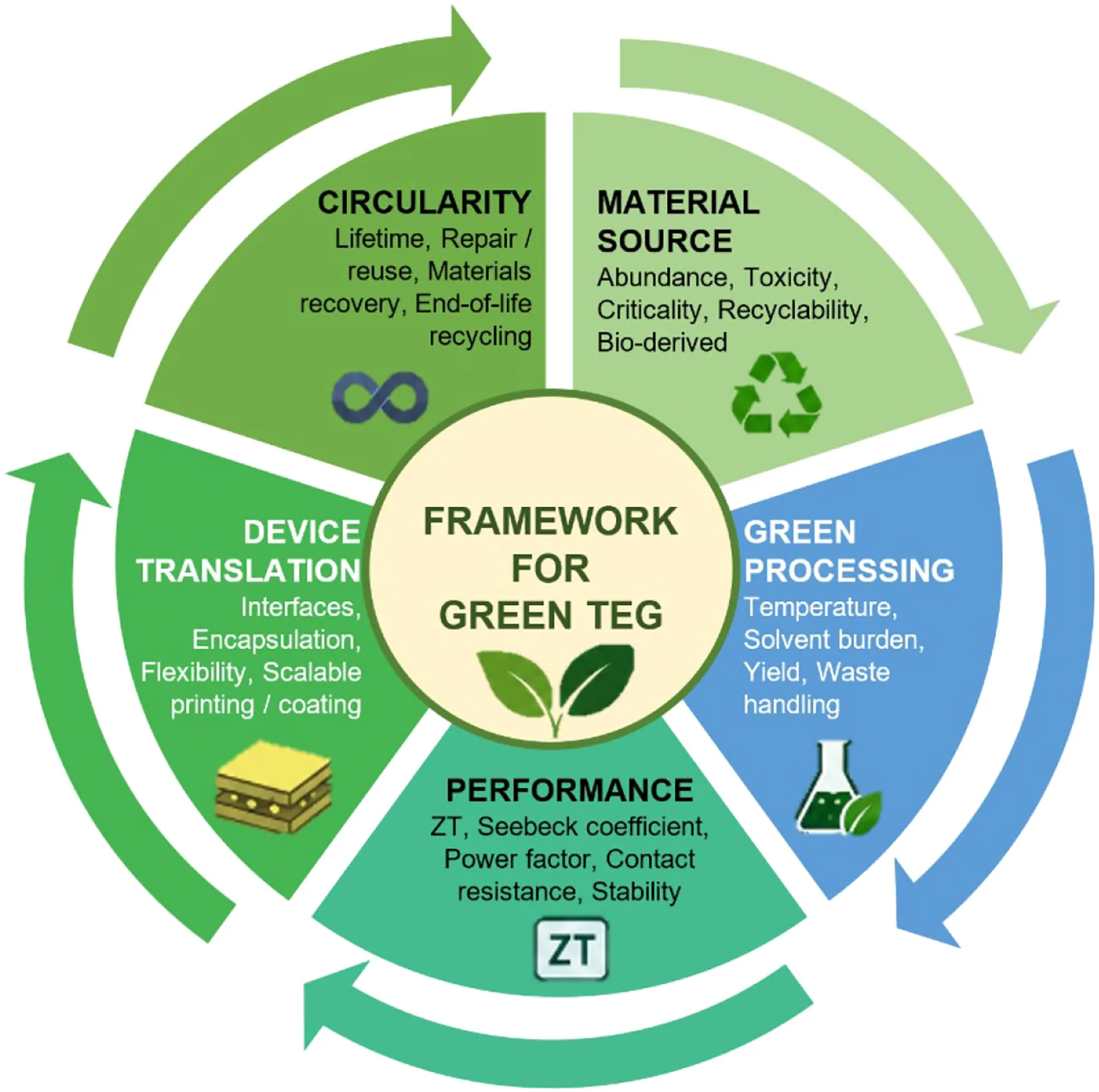
Proposed evaluation framework for green thermoelectric generators Green TEG claims should connect material source, processing route, device performance, device translation, and circular outcome through a minimum reporting set rather than relying on a single green precursor or a single performance metric.

In this perspective, we treat green thermoelectric design in a few viewpoints. First, the active material should be assessed for abundance, toxicity, recyclability, and functional stability. Then, the synthesis route should be reviewed through temperature, solvent burden, energy use, water demand, yield, and waste handling. Next, the performance should include not only *ZT* and power factor but also output power, power density, contact resistance, and stability under realistic temperature gradients. Importantly, device design must account for thermal interfaces, encapsulation, flexibility, and manufacturability. Finally, environmental claims should be assessed through life cycle thinking rather than inferred from the use of one green precursor.

To substantiate the proposed framework, two contrasting systems are examined: a PEDOT:PSS device processed via vitamin C-assisted aqueous synthesis,^5^ and recycled Bi_2_Te_3_ system derived from wafer-cutting waste.^6^ The PEDOT:PSS system scores favorably on material source and process energy, with abundant precursors, a benign reducing agent, and near-ambient aqueous processing. Its performance is modest but sufficient for wearable and flexible applications (electrical conductivity of 400 Scm^−1^, Seebeck coefficient of 30 μVK^−1^, resulting in a power factor of 51.8 μWm^−1^K^−2^). Circularity is partially addressed through water-soluble processing, though end-of-life recovery protocols remain underdeveloped. By contrast, the recycled Bi_2_Te_3_ system scores highly on performance (*ZT* > 1.0 with proper impurity management) and device maturity, which draws its circularity advantage from a waste-derived feedstock. However, purification and sintering add process energy, and acid leaching introduces secondary waste streams that must be responsibly managed. Conventional *ZT*-centric evaluation would rank these systems far apart, but the proposed framework reveals a more subtle view in which each excels on different dimensions, and the preferred choice depends on the target application rather than any single metric.

It should be noted that this article is not a comprehensive review of all thermoelectric materials. Instead, it discusses how green fabrication routes and green-source materials affect the structure-property relationships that control thermoelectric performance. The discussion covers performance metrics, fabrication routes, material classes, device progress, and practical directions needed for sustainable and scalable low-grade heat harvesting.

## Thermoelectric performance metrics and materials design considerations

Thermoelectric conversion is usually discussed through the Seebeck, Peltier, and Thomson effects.^7^^,^^8^ For power generation, the Seebeck effect is the focal point. When a temperature difference is applied across a material or junction, charge carriers diffuse from the hot side to the cold side and generate an open-circuit voltage. The Seebeck coefficient is defined as *S = ΔV/ΔT*, where Δ*V* is the generated voltage and Δ*T* is the temperature gradient.^9^ Metals generally have small S, whereas semiconductors can produce much larger values and are therefore preferred for practical thermoelectric applications.^10^ Because *S* depends on temperature and carrier concentration, reported values should always be linked to the measurement temperature and material state.

In a practical TEG, p-type and n-type terminals are connected electrically and placed between a heat source and a heat sink. The material-level figure of merit is *ZT = S*^*2*^*σT/κ*, where *S* is the Seebeck coefficient, *σ* is electrical conductivity, T is absolute temperature, and κ is total thermal conductivity.^11^ The term *S*^*2*^*σ* is the power factor while κ contains electronic and lattice contributions, *κ = κ*_*e*_ *+ κ*_*latt*_. Electrical conductivity depends on carrier density and mobility. The term can also be written as *σ = e(nμ*_*e*_ *+ pμ*_*h*_*)*, where e is the elementary charge, n and p are electron and hole concentrations, respectively, and *μ*_*e*_ and *μ*_*h*_ are the corresponding mobilities. These parameters are coupled. Increasing carrier concentration can improve *σ*, but it often reduces S and increases the electronic part of thermal conductivity. Reducing *κ* is also not straightforward, because aggressive defect formation or nano-structuring may scatter phonons while also reducing carrier mobility. Much of modern thermoelectric materials design has therefore focused on selective phonon scattering i.e., reducing lattice thermal conductivity while preserving electronic transport through band engineering, defect control, grain-boundary design, and nanoscale interfaces.^12^^,^^13^^,^^14^^,^^15^^,^^16^

Two strategies have achieved prominence in addressing the *S - σ - κ*_*latt*_ trade-off. Firstly, band convergence i.e., engineering multiple valence or conduction band valleys to coincide in energy that increases the Seebeck coefficient without a proportional loss of carrier mobility, a principle first demonstrated in Na-doped *PbTe*_*1-x*_*Se*_*x*_.^17^ Notably, this strategy has already been demonstrated within a green-compatible synthesis route: In/Pb co-doped *SnTe* prepared via green hydrothermal synthesis achieved enhanced performance through simultaneous band convergence and resonant-level formation.^18^ Band convergence is not universally beneficial, however, and its efficacy depends on the strength of interband carrier scattering, which must be evaluated on a system-specific basis.^19^ Entropy engineering offers a complementary route, introducing high configurational entropy through multi-principal-element alloying to suppress lattice thermal conductivity via severe phonon scattering while simultaneously stabilizing desired crystal structures.^20^ Both strategies represent productive directions for coupling thermoelectric performance gains with green-compatible material design, and their systematic application to sustainable material systems remains a promising area for future work.

Meanwhile, device efficiency depends on the available temperature gradient. The maximum conversion efficiency can be computed from *η*_*max*_ *= [(T*_*h*_ *- T*_*c*_*)/T*_*h*_*] × [((1 + ZT*_*avg*_*)*^*1/2*^ *- 1)/((1 + ZT*_*avg*_*)*^*1/2*^ *+ T*_*c*_*/T*_*h*_*)]*, where *T*_*h*_ and *T*_*c*_ are the hot- and cold-side temperatures, respectively, and *ZT*_avg_ is the average figure of merit across the operating range.^11^^,^^21^ This expression shows why high material performance alone is not enough. A device operating at a small *ΔT* may produce little useful power even if the material has an attractive power factor.

For green thermoelectrics, conventional performance metrics must be retained but not treated as the only success criteria. Low-temperature synthesis, water-based processing, biodegradable substrates, and waste-derived precursors can narrow the range of available processing tools. They may also introduce impurities, microstructural heterogeneity, lower crystallinity, or unstable dopant states. These features should not be regarded merely as drawbacks, but rather as inherent aspects of the design space. Defects, interfaces, porous structures, and percolating networks can suppress heat transport while maintaining useful charge transport, but only when they are deliberately controlled. The central design question is therefore not whether the material is “green”, but whether the green synthesis route can deliver reproducible materials and devices with meaningful power output and reduced overall environmental impact.

## Green fabrication methods

The sustainability value of a thermoelectric material depends on how it is manufactured. Conventional thermoelectric processing often uses high-temperature melting and processing, vacuum deposition, strong acids, or hazardous organic solvents. These methods can give excellent control over composition and crystallinity, but they can also impact the environmental footprint of the final device. A green fabrication route should therefore be judged by more than the identity of the solvent. Energy demand, precursor toxicity, yield, post-treatment, waste generation, scalability, and end-of-life options must all be considered.

Water-based processing is the most direct improvement over solvent-centric routes. Aqueous doped carbon nanotube (CNT) and polymer systems have been reported to reduce volatile organic solvent consumption while remaining compatible with coating, printing, and flexible substrates, with device performances comparable to those of conventional systems.^2^^,^^5^ Despite these advantages, some limitations remain. Surfactants, dopants, washing steps, drying energy, and wastewater treatment can still influence the final environmental burden. Water-based processing should therefore be seen as a useful starting point toward sustainable processing rather than a complete solution claim.

Separately, bio-assisted and biomass-derived synthesis offers an attractive route. Plant extracts, sugars, lignin, cellulose, and other naturally derived compounds can act as reducing agents, stabilizers, templates, or carbon sources.^5^^,^^22^ The advantage lies not only in their renewable origin but also in their ability to generate hierarchical morphologies, porous structures, and tailored interfacial chemistry under mild conditions, which eliminate the need for hazardous agents. Such structures are highly attractive for thermoelectric applications because they can potentially decouple electrical and thermal transport by enhancing phonon scattering while preserving carrier mobility. Unfortunately, their weakness lies in reproducibility. Natural feedstocks vary by source, season, purification method, and composition. Industrial translation will require tighter control of yield, impurity level, and batch-to-batch performance.

Hydrothermal synthesis is often described as “green” processing because water is used as the reaction medium and the synthesis temperature is usually lower than melting or solid-state routes.^18^^,^^22^^,^^23^^,^^24^^,^^25^ The process allows for control over nucleation, morphology and defect chemistry. However, sealed reactors, long reaction times, drying steps, acidic precursors, and washing procedures can offset part of the environmental benefits. Hydrothermal routes should therefore be compared using process-level data like energy demand and chemical footprint, rather than labeled “green” by default.

Electrochemical deposition is especially relevant for thin-film and flexible devices. It operates at low temperature, can be carried out in aqueous or mild electrolytes, and allows control over film thickness and composition.^26^^,^^27^^,^^28^^,^^29^^,^^30^^,^^31^^,^^32^ Both n-type and p-type Bi-Sb-Te films have been successfully fabricated using this method.^32^ These features make it more compatible with scalable manufacturing than many vacuum-based methods. Despite these advantages, careful management of metal-containing electrolytes, hydrogen evolution, pH control, and electrolyte disposal remain essential. Accordingly, closed-loop electrolyte recovery and metal-ion recycling should be embedded within the process architecture from the outset rather than implemented retrospectively.

Printing, spray coating, and related additive methods are useful too because they can reduce material waste and allow large-area or roll-to-roll fabrication. Their sustainability depends on ink chemistry. A printed device is not necessarily “green” if it uses persistent binders, hazardous solvents, or requires high temperature annealing after deposition. For flexible green TEGs, the most convincing route will combine benign inks, low-temperature curing, stable interfaces, and recoverable or biodegradable substrates.

Laboratory-scale demonstrations of green fabrication frequently underestimate the environmental burden that emerges at larger production volumes. Water-based and additive manufacturing routes are best positioned to retain their sustainability advantages upon scale-up, particularly where roll-to-roll compatibility can be achieved. Hydrothermal synthesis loses its environmental edge rapidly: pressurized reactors at pilot and industrial scale demand substantially more energy, generate larger volumes of acidic or alkaline waste, and require more demanding reactor infrastructure. Electrochemical deposition scales reasonably for thin-film applications but faces disproportionate increases in metal-containing electrolyte volume and replenishment frequency, making closed-loop electrolyte recovery an economic necessity rather than an optional practice. Bio-assisted synthesis presents the most severe scalability challenges, with batch-to-batch feedstock variability, purification demands, and low volumetric yield likely restricting this route to niche or high-value applications in the near term. These trade-offs are summarized in Table 1.

**Table 1:** Comparative sustainability and life cycle considerations of green thermoelectric fabrication routes

Route	Main burden to report	Energy/process intensity	Scalability outlook	Scalability risk to sustainability	LCA priority
Water-based processing	surfactant disposal, wastewater, drying energy	low	high for coating and printing	low	water treatment, additive recovery, drying load
Bio-assisted synthesis	batch variability, low yield, purification	low to moderate	moderate	high	biomass sourcing, purification, batch consistency
Hydrothermal/solvothermal synthesis	pressurized reactors, acids, solvents, washing	moderate to high	moderate	high	reaction duration, solvent recovery, wastewater
Electrochemical deposition	electrolyte disposal, metal-ion waste, gas evolution	low	high for thin films	moderate	electrolyte recycling and metal recovery
Additive manufacturing	ink solvent, binder persistence, curing step	low to moderate	high for roll-to-roll	low to moderate	ink formulation and end-of-life separation
Recycled inorganic processing	impurity removal, acid leaching, secondary waste	moderate	high if closed-loop recovery is built in	moderate	recovery efficiency, purification energy, circularity

As green thermoelectric research continues to expand, it has become increasingly clear that sustainability cannot be evaluated solely based on the use of renewable precursors or water-based solvents. A more rigorous assessment requires life cycle analysis (LCA), which examines environmental impacts across the full material and device life cycle, including raw material extraction, synthesis, processing, operation, and end-of-life disposal or recycling. In thermoelectric systems, key LCA indicators include energy consumption, greenhouse gas emissions, solvent toxicity, water usage, waste generation, recyclability, and resource criticality.

For example, biomass-derived carbon materials are generally viewed as environmentally favorable because they utilize renewable feedstocks and support waste valorization. However, high-temperature activation or graphitization processes can substantially increase embodied energy and carbon emissions. Similarly, hydrothermal synthesis reduces reliance on volatile organic solvents but may still involve prolonged heating and pressurized reactors that contribute significantly to process energy demand. Electrochemical deposition offers low-temperature processing advantages, although electrolyte management and metal-containing wastewater treatment remain important environmental considerations.

Soleimani et al.^1^ provided a comprehensive life cycle comparison of different thermoelectric systems. They examined inorganic (Bi_2_Te_3_-based), organic (PEDOT:PSS-based), and hybrid systems across multiple impact categories. They observed that inorganic thermoelectric systems carry substantially higher embodied energy and global warming potential per unit of functional output compared to organic alternatives. These are driven primarily by raw material extraction and high-temperature processing. On the other hand, PEDOT:PSS-based systems show lower cradle-to-gate greenhouse gas emissions although solvent use in certain processing routes remains a concern. Hybrid systems occupy an intermediate position. These findings support the central argument of this perspective, that carbon footprint and embodied energy can differ by an order of magnitude across thermoelectric material families, and that this difference must be incorporated into design decisions alongside *ZT*. But they also illustrate a broader limitation: a fully harmonized, ISO 14040/14044-compliant comparison across all green material classes is not yet possible, as published LCA studies in this field (including Soleimani et al.^1^) differ in system boundaries, functional units, and impact assessment methods. Future studies should adopt consistent, ISO-based functional units to enable meaningful cross-comparison. Until such harmonized data exist, comparative sustainability claims in this field, including those made here, should be treated as directional rather than precise. Thus, these examples show that no fabrication route is inherently sustainable in isolation; rather, sustainability depends on balancing thermoelectric performance with environmental and economic impacts throughout the entire material life cycle.

Future development of green thermoelectric fabrication should prioritize low-temperature processing (<300°C), renewable or bio-derived precursors, scalable additive manufacturing, closed-loop solvent recovery, and improved end-of-life recyclability. Critically, standardized LCA methodologies should be incorporated alongside thermoelectric characterization to enable meaningful cross-comparison of material systems and processing routes; since moderate-performance materials processed under low-energy conditions may offer greater net sustainability than high-performance alternatives requiring energy-intensive synthesis or scarce elements. Integrating these green fabrication principles with high-performance material design will ultimately be essential for translating laboratory advances into environmentally responsible and commercially viable thermoelectric technologies. Key LCA considerations for representative fabrication routes are summarized in Table 1. To enable meaningful cross-comparison of green thermoelectric systems, we propose the parameters listed in Table 2 be reported alongside thermoelectric performance data as a minimum standard for green thermoelectric assessment. This checklist is not intended to replace full LCA, which remains the most rigorous method. Rather, it provides a rapidly accessible set of parameters that can be collected during routine synthesis and device characterization without dedicated LCA infrastructure. Adoption of this checklist as a community norm would significantly improve the comparability of green thermoelectric claims across research groups and institutions.

**Table 2:** Proposed minimum reporting checklist for green thermoelectric fabrication routes

No.	Parameter	Reasons for evaluation
1	synthesis temperature	proxy for process energy demand and compatibility with temperature-sensitive substrates.
2	processing time	total thermal and/or reaction time, which contribute to the cumulative energy consumption.
3	solvent	important for toxicity classification, waste volume, and recovery burden.
4	precursor	source classification: natural/synthetic, recycled/recovered, or bio-derived, with geographic origin where relevant.
5	yield	resource efficiency of the synthesis route: low yield implies higher per-mass material and waste burden.
6	post-treatment processes	identity of any annealing, washing, acid treatment, or surface functionalization and associated conditions that can contribute to the above parameters.
7	waste	waste type (solid, liquid, gaseous) and estimated volume or mass per mass of product.
8	end-of-life	whether material recovery, biodegradation, or safe disposal has been demonstrated (yes/no and method).
9	device lifetime	operational hours or thermal cycles under specified conditions.

The fabrication routes discussed previous show how a thermoelectric material is processed, but the overall sustainability profile of a TEG system is equally shaped by what the material is made from. Green processing applied to scarce or toxic precursors offers limited net benefit. Conversely, abundant, and bio-derived materials processed through energy-intensive or solvent-heavy routes may also fall short of genuine sustainability. The evaluation framework introduced in Section 1 therefore requires that both process and source dimensions be addressed simultaneously. The following section examines the four principal classes of green-source thermoelectric materials in terms of their thermoelectric function, supply chain characteristics, abundance, and end-of-life options.

## Green-source thermoelectric materials

Beyond fabrication routes, the sustainability of thermoelectric systems is fundamentally linked to material sourcing. Conventional inorganic thermoelectrics such as *Bi*_*2*_*Te*_*3*_, *PbTe*, and related chalcogenides remain difficult to replace when the only criterion is *ZT*. Green-source materials are usually weaker on peak performance, but stronger in flexibility, abundance, processing temperature, biocompatibility, or circularity. Four broad classes are especially relevant: bio-polymer and organic systems, biomass derived or waste-derived carbon materials, recycled inorganic thermoelectrics, and ionic thermoelectric systems.

The supply-chain risk associated with conventional thermoelectric materials is substantial. Tellurium, the key element in *Bi*_*2*_*Te*_*3*_ and *PbTe*, has a crustal abundance of approximately 1 ppb, making it one of the rarest stable elements on Earth. Approximately 90% of global tellurium supply originates as a by-product of copper refining, creating structural supply-chain dependency and geographic concentration. Bismuth is more available (∼9 ppb crustal abundance) but similarly by-product dependent. Bismuth is classified under the European Union Critical Raw Materials framework, which identifies supply risk based on economic importance and supply concentration metrics. In contrast, the primary input elements for representative green alternatives (i.e., carbon [biomass derived], copper iodide [*CuI*], and the PEDOT:PSS precursor) are substantially less constrained in terms of abundance and supply geography, though copper itself carries moderate criticality. Incorporating material criticality metrics alongside ZT in comparative assessments is therefore not merely an academic exercise; it directly informs the long-term scalability and geopolitical resilience of thermoelectric technology.

This geographic concentration also creates a structural geopolitical dependency: export restriction, trade policy shifts, or conflict affecting the small number of refining nations involved could constrain Bi_2_Te_3_ availability and cost independent of any technical or environmental factor. Demand from competing sectors, such as CdTe photovoltaics, adds further pressure to an already thin tellurium supply chain. Green material systems built on carbon, copper, and biomass-derived precursors draw on more geographically distributed supply chains, making supply-chain resilience a further argument for their long-term sustainability, and one that should be treated as a design criterion rather than an implicit assumption.

### Bio-polymer and organic thermoelectric materials

Organic thermoelectric materials include synthetic conductive polymers and bio-derived polymer systems. PEDOT:PSS, polyaniline, and polypyrrole have been studied widely because they are solution processable, mechanically compliant, and chemically tunable.^33^^,^^34^^,^^35^ Besides, their performance can be improved through secondary doping, solvent treatment, carbon nanofillers, and hybridization with inorganic phases. For flexible and wearable systems, these advantages can matter as much as peak ZT.^36^^,^^37^ The limitation of these material systems is that most conductive polymers still suffer from modest long-term stability and a difficult trade-off between conductivity and Seebeck coefficient. PEDOT:PSS-based composites with tetrahedrite nanoparticles, for example, show that hybridization can improve transport and reduce thermal conductivity, but the inorganic fraction, sintering condition, and filler distribution determine whether the system remains genuinely sustainable.^38^ Bio-polymers such as cellulose, chitin, starch, proteins, and lignin offer biodegradability, low toxicity, and renewable sourcing.^39^^,^^40^ On their own, however, they are usually too electrically insulating for direct thermoelectric application. Their more realistic role is as substrates, binders, matrices, ion conductors, or interfacial modifiers. When combined with CNTs, graphene, CuI, or other conductive phases, they can provide mechanical support and environmental value while the conductive network carries charge.^37^^,^^41^^,^^42^^,^^43^^,^^44^ Lignin-CNT systems demonstrate this potential: functionalized lignin can tune n-type or p-type behavior and improve energy filtering at interfaces.^45^^,^^46^ At the same time, some high-performing lignin/CNT reports still rely on solvents such as NMP or on non-renewable nanofillers. These systems should therefore be described as partly bio-derived or bio-assisted rather than fully green unless solvent recovery, filler sourcing, and end-of-life recovery are addressed. Biomass feedstock availability is geographically variable, and sustainability claims based on renewable sourcing should be regionally contextualized. Rice husk-derived carbons are most viable in major rice-producing regions of Southeast Asia and parts of Africa; lignin availability follows the distribution of kraft pulping industries; and cellulose quality varies significantly by species and agricultural practice. A material that is genuinely low-burden in one regional context may require energy-intensive transportation or substitution in another. This regional specificity should therefore be acknowledged as a practical constraint in life cycle assessments and technology transfer planning rather than treated as a blanket endorsement of bio-derived routes. Bio-polymer and organic composites should not be benchmarked against conventional inorganic thermoelectrics on ZT alone. Their real comparative value emerges in device-level evaluation, where flexibility, biodegradability, low-temperature processability, and form-factor compatibility collectively define the application outcomes. We assert that the primary bottleneck for this class is not intrinsic Seebeck coefficient but long-term doping stability under ambient humidity and thermal cycling. Addressing this challenge is likely to be more commercially impactful than further optimizing peak power factor in pristine films.

### Carbon-based materials from green sources

Biomass and waste products can be converted into graphitic carbon, carbon dots, reduced graphene oxide-like materials, and porous carbon frameworks.^47^^,^^48^^,^^49^ Their appeal is clear: carbon is abundant, chemically stable, mechanically robust, electronically tunable and compatible with flexible or disposable devices. Simple demonstrations such as graphite pencil traces on paper also show that very low-energy fabrication can generate measurable thermoelectric voltages.^50^^,^^51^ The main weakness of this material system is transport coupling. Highly conductive carbon networks often also conduct heat well. Strategies such as defect creation, porosity, heteroatom doping, and composite formation can reduce thermal conductivity, but these modifications may simultaneously lower carrier mobility. Biomass-derived carbon further raises a process concern: activation and graphitization require high temperatures, chemical activators, and washing steps. While the precursor itself is renewable, the overall process may not be low burden unless these steps are controlled. The best near-term role for biomass-derived carbon is therefore not to compete directly with optimized *Bi*_*2*_*Te*_*3*_ modules. Instead, it is more likely to find application in low-cost, flexible, lightweight, or disposable energy-harvesting platforms, particularly where small power output is acceptable, and the device can be made from low-value waste. Continued progress will depend on controlling pore structure, dopant chemistry, and interfaces so that electrical pathways are preserved while heat transport is reduced. The research community has devoted considerable effort to optimizing Seebeck coefficient and electrical conductivity in biomass-derived carbons. We contend, however, that the more consequential engineering challenge is the decoupling of thermal conductivity. Carbon networks that are sufficiently conductive for thermoelectric use tend to be equally efficient at transporting heat, limiting *ZT* improvement. Structural strategies such as controlled porosity, heteroatom doping, and composite interfaces, must therefore be prioritized alongside precursor selection if this class of materials is to progress beyond niche demonstrations.

### Inorganic thermoelectric materials from waste and recycled sources

Recycled and waste-derived inorganic thermoelectrics offer the highest performance ceiling among green-source options. Rather than replacing inorganic thermoelectrics entirely, this route investigates whether useful materials can be recovered from cutting waste, spent modules, photovoltaic waste, agricultural residues, or industrial side streams.^52^ Rice husk, for example, contains silicon-rich components that can be converted into SiC or Mg_2_Si-based materials.^53^^,^^54^ *Bi-Te* intermetallic alloys from photovoltaic manufacturing have also been explored as a source for thermoelectric materials.^55^ The primary advantage of recycling lies in lowering the demand for virgin raw materials. *Bi*_*2*_*Te*_*3*_ wafer-cutting powders and tellurium-containing waste can retain valuable thermoelectric potential if impurities, oxides, and carbon residues are managed properly.^6^ Bio-leaching and other lower-temperature recovery methods may reduce the burden of metal extraction too.^56^

Nonetheless, the sustainability claim must be justified. Acid leaching, solvent usage, purification, reactor corrosion, metal-containing wastewater, and secondary waste can all diminish the environmental advantage. Recycled inorganic systems should therefore be reported together with recovery yield, purity, energy input, and waste-treatment requirements. Among green thermoelectric routes, this class is likely to be most relevant for applications that need higher output power, but only if closed-loop recovery is integrated into the device life cycle. In our assessment, recycled inorganic thermoelectrics represent the most direct route to high-performance green TEGs, yet the field has underinvested in closed-loop process design. Most published recycling demonstrations treat recovery as a one-time extraction from a specific waste stream rather than as a designed circularity architecture. We propose that the next meaningful step is integrating recovery yield, purity metrics, secondary waste generation, and energy input into device-level reporting as standard practice, making the full circular benefit or its absence, transparent.

### Ionic thermoelectric materials

Ionic thermoelectric materials are an entirely different class of thermoelectric system. They are not direct equivalents of conventional solid-state electronic thermoelectrics; rather, they constitute a related category of low-grade heat-harvesting systems in which ion migration or redox reactions generate a thermovoltage. Their appeal lies in Seebeck coefficients that can reach the millivolt-per-kelvin range, far exceeding typical electronic thermoelectrics (Figure 2).^57^ However, they share common drawbacks, including low power density, slow response, liquid or gel stability, leakage risk, and electrode/electrolyte durability. Two distinct mechanisms underlie ionic thermoelectric behavior. In thermodiffusive ionic systems, a temperature gradient drives unequal cation and anion migration, leading to charge separation at interfaces. This differential migration results in an imbalance in ion concentrations across the material which can be explained with the Gouy-Chapman-Stern theory. In thermogalvanic cells, by contrast, temperature-dependent redox reactions generate an electrochemical potential difference.

**Figure 2:**
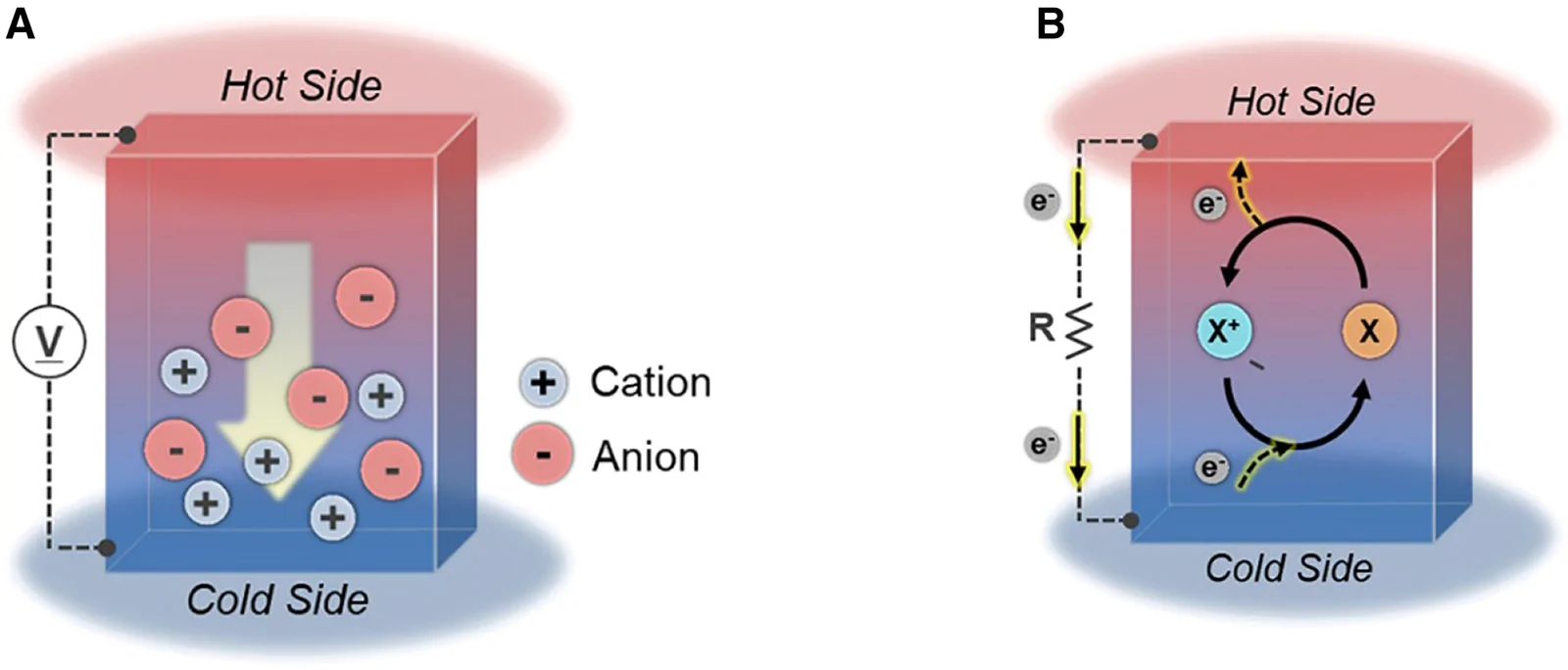
Ionic thermoelectric (A) Thermodiffusion effect. (B) Thermogalvanic effect. Reproduced from.^57^ 2025 CC BY.

On the other hand, in thermodiffusive ionic systems, aqueous systems are attractive from an environmental aspect. However, water can screen ionic interactions and limit the thermopower.^58^ Organic solvents and polymer matrices may increase thermopower via ion solvation setting, although often at the expense of ionic conductivity or environmental simplicity.^59^^,^^60^ Recent strategies such as nanochannel confinement, cellulose membranes, lignin-derived hydrogels, and all-wood ionic gels have demonstrated how bio-derived structures can improve ion selectivity and thermopower.^61^^,^^62^^,^^63^^,^^64^ Meanwhile, thermogalvanic systems based on ferri/ferrocyanide or *Fe*^*2+*^*/Fe*^*3+*^ redox couples have also been improved through solvent engineering, entropy modulation, and gel electrolytes.^65^^,^^66^^,^^67^^,^^68^^,^^69^^,^^70^^,^^71^^,^^72^ Bio-inspired double-layer thermogalvanic cells further show that spatial separation of redox pairs can reduce ion pairing and enhance output.^73^ The relationship between ion concentration, ionic conductivity, and thermopower is more complex than in electronic thermoelectrics. Increasing carrier concentration can improve ionic conductivity and boost the thermovoltage, though as in electronic thermoelectrics, *S* and *σ* are generally coupled rather than independently tunable, meaning gains in power factor (*S*^*2*^*σ*) typically involve a similar trade-off to that discussed in thermoelectric performance metrics and materials design considerations.^74^^,^^75^ For this reason, ionic thermoelectrics should be judged by device-level voltage, power density, response time, cycling stability, leakage resistance, and electrode compatibility.

Despite their attractive thermopower, the environmental profile of ionic thermoelectric systems requires explicit scrutiny that is frequently absent from performance-focused reports. Ferri/ferrocyanide ([Fe(CN)_6_]^3-/4-^) remains among the most widely studied thermogalvanic redox couples, yet cyanide-containing species raise aquatic toxicity concerns if released through device leakage, electrode degradation, or improper end-of-life disposal. Many high-thermopower gel electrolytes rely on polymer matrices with limited or condition-dependent biodegradability (e.g., polyacrylamide, or polyvinyl alcohol, whose degradation requires specific microbial environments) or on organic co-solvents with uncertain long-term environmental persistence. Cellulose- and lignin-derived hydrogels represent genuinely greener alternatives,^61^^,^^62^^,^^63^ but their thermoelectric performance remains lower and their durability under repeated thermal cycling is less established. Moving toward truly safe-by-design ionic thermoelectrics requires deliberate component choices, including aqueous electrolytes based on environmentally benign redox couples (such as Fe^2+^/Fe^3+^ sulfate systems), biodegradable gel matrices, and leakage-resistant device encapsulation that permits electrolyte recovery at end of life. Reporting toxicity classifications and biodegradability data for electrolyte components should become a standard expectation in this subfield, equivalent in importance to reporting the Seebeck coefficient. Across all four classes, the same fundamental tension consistently reappears in different forms. Table 3 contextualizes green material performance against conventional benchmarks. Recycled inorganic materials deliver the strongest performance but demand careful environmental stewardship. Organic and bio-derived systems provide flexibility and biodegradability yet remain constrained by intrinsic transport limitations. Ionic thermoelectrics exhibit impressive Seebeck coefficients but continue to face challenges in power density and long-term stability. Whether these green materials can realistically compete with conventional systems is therefore application-dependent. Displacement of optimized *Bi*_*2*_*Te*_*3*_ or *PbTe* in high-output industrial heat recovery or precision cooling is unlikely in the near term. By contrast, in wearable body-heat harvesting, flexible distributed sensing, low-power IoT supply, and disposable medical or environmental monitoring, where the required output falls in the microwatt-to-milliwatt range and where weight, conformability, biocompatibility, or toxicity constraints matter, green materials are already competitive or approaching competitiveness. The framework proposed here is designed to identify these application niches with precision, making visible the dimensions on which green materials can prevail even when their peak *ZT* is lower. Looking ahead, the most promising strategies will likely involve hybrid architectures and circular-economy approaches that treat environmental sustainability and competitive performance as complementary rather than conflicting design objectives.

**Table 3:** Comparative summary of green thermoelectric material classes across performance, processing, life cycle, and sustainability indicators

Material class	ZT (temp. Range)	Power factor (μWm^−1^ K^−2^)	Processing compatibility	Material source/criticality	Recyclability	Notable features
Bi_2_Te_3_ (benchmark)	1.5–1.8 (300–400 K)	4,000–5,000	high-temp, sintering; vacuum processing	critical - Te (∼1 ppb crustal abundance); byproduct-dependent supply	low; energy-intensive recovery; no closed-loop design	proven commercial applications with optimized modules
Bio-polymer and organic composites	0.1–0.4 (300 K)	100–1,000	aqueous; low-temp; solution-processable; flexible substrate compatible	non-critical -abundant PEDOT precursors; bio-derived fillers available	moderate; water-processable; doped-state recovery underdeveloped	flexible, printable, compatible with soft substrates. low ZT, unstable doping, humidity sensitivity
Biomass-derived and waste-derived carbon	0.01–0.1 (300 K)	10–100	hydrothermal; pyrolysis; moderate temp.	non-critical - regionally variable feedstock (rice husk, lignin, cellulose)	high; biodegradable substrates; carbon source recoverable	abundant feedstocks, low cost, circularity value. low efficiency and difficult *σ-κ* decoupling
Recycled inorganic thermoelectrics	0.8–1.5 (300–500 K)	2,000–4,000	purification + sintering; moderate energy	moderate - waste-derived; reduces virgin Te/Bi demand	moderate-high; closed-loop viable if designed in	highest performance potential among green routes.purification burden, metal waste, element criticality
Ionic thermoelectrics	N/A^a^	low	aqueous gel casting; low-temp; flexible	non-critical-aqueous electrolytes; gel matrices	variable; biodegradable gels possible; redox couple recovery limited	high thermopower and soft material compatibility. low power density, leakage, slow response, stability

Conventional Bi_2_Te_3_ is included as a benchmark.

a Note: Ionic thermoelectrics are characterized by Seebeck coefficient rather than ZT, as the ionic/electronic transport coupling makes direct ZT comparison with electronic thermoelectrics inappropriate.

## TEG device progress and outlook

Translating green thermoelectric materials into practical devices remains a major challenge. High Seebeck coefficients or power factors measured in small-scale films do not necessarily translate into useful module-level power output, as material-level metrics alone cannot capture the full complexity of real device configurations. The following discussion addresses key device-level considerations i.e., from interfacial losses and module engineering to long-term operational stability, that determine whether laboratory-demonstrated green materials can become deployable technologies.

### Device demonstrations and practical applications

Recent progress nevertheless demonstrates the feasibility of environmentally responsible TEG devices. Nanocellulose-based substrates derived from renewable biomass have emerged as promising platforms for flexible thermoelectrics. CuI/nanocellulose composites have shown Seebeck coefficients and power factors suitable for low-temperature operation, while lightweight CuI/nanocellulose modules generate useful power output from body heat and small industrial temperature gradient.^76^^,^^77^ These examples confirm that green material choices and competitive device performance are not mutually exclusive. Wearable TEGs further illustrate the importance of device-level engineering. In body-heat harvesting systems, the available temperature gradient is typically small and highly dynamic. Device output therefore depends not only on intrinsic material properties, but also on convective cooling, skin contact, textile integration, bending, and motion. Reports showing higher output during walking than at rest highlight how mechanical coupling influences thermal management in wearable configurations.^78^ Achieving reliable and consistent output under these conditions requires co-design of the material, substrate, electrode geometry, and encapsulation from the outset.

Carbon- and polymer-based devices present similar trade-offs. Carbon nanotubes and graphene provide high electrical conductivity and mechanical flexibility, but their large-scale production can still be energy intensive. Conductive polymers such as PEDOT:PSS are attractive because of their printability and compatibility with flexible substrates, although their ZT values remain modest and doped states are sensitive to humidity and thermal cycling. Progress in this area increasingly depends on device architecture and interface engineering rather than further optimization of the bulk material alone. For example, biomass-derived carbons, including coffee-ground-derived carbon dots, offer stronger circular-economy potential, but challenges remain in controlling composition, conductivity, and device reproducibility.^79^

### Interfacial and contact engineering

Contact resistance and thermal interface losses are among the most consequential and under-reported factors determining practical performance in green thermoelectric devices. Intrinsic ZT or power factor values measured on free-standing films or pellets often fail to account for the significant voltage drops and thermal short-circuits that arise at electrode contacts and material interfaces in real module configurations. This discrepancy is particularly pronounced in printed, gel-based, and bio-derived systems, where the electrode-material interface is often soft, chemically mismatched, or geometrically irregular. Specific contact resistance values below 10 μΩ cm^2^ are typically required for competitive module efficiency, whereas values an order of magnitude higher are common in unoptimized flexible device architectures. Interface engineering strategies compatible with green fabrication constraints include conductive adhesives with low curing temperatures, PEDOT:PSS or CuI interlayers that improve wetting and charge injection at organic-inorganic interfaces, surface treatments that reduce native oxide barriers without introducing hazardous reagents, and *in-situ* sintering approaches for nanostructured contacts. Thermal interface resistance at the substrate level is equally critical. Flexible polymer substrates improve mechanical conformability but can simultaneously act as thermal insulators, reducing the effective temperature gradient across the active material. Reporting contact-specific resistance alongside material-level metrics should therefore be treated as a minimum requirement for device-level publications in this field.

A natural question is whether interface engineering or continued bulk material optimization offers the larger practical efficiency gain. For unoptimized flexible or printed systems, contact and thermal interface resistance frequently account for a comparable or larger share of total module efficiency loss than incremental gains in bulk ZT, since many green materials already sit in a regime of diminishing returns on further material optimization. Additionally, poor interfacial contacts introduce voltage losses and thermal short-circuits that compound across the many thermocouple legs in a module rather than adding linearly. For early-stage green TEG systems, interface optimization is therefore likely to deliver a more immediate and cost-effective efficiency gain than further material optimization alone, though both must ultimately be pursued in parallel as material performance matures.

### Module-level engineering

Translating green materials into functional modules introduces engineering challenges that are distinct from, and in some ways more demanding than those faced with conventional inorganic systems. Several challenges deserve attention.

#### n-type/p-type pairing imbalance

The most persistent module design challenge for green thermoelectrics is the asymmetry between available p-type and n-type materials. PEDOT:PSS, most bio-polymer composites, and the majority of carbon-based materials are inherently p-type. High-performance n-type green alternatives remain scarce, forcing module designers to accept inferior performance or combine green p-type legs with conventional inorganic n-type legs. Lignin-functionalized CNT systems have shown promise for n-type conversion,^45^ but stability under operational conditions requires further validation.

#### Thermal management

Flexible and bio-derived substrates that confer mechanical compliance often act as thermal insulators, reducing the temperature gradient that drives device output. Module geometries must therefore balance thermal distribution, substrate thickness, and thermocouple leg aspect ratio to maintain useful ΔT across the active material.

#### Encapsulation and circularity

Encapsulation improves device durability against humidity and oxidation, but conventional encapsulation materials such as epoxies and barrier films can compromise end-of-life material recovery. Encapsulation approaches that use thermoplastic or solvent-dissolvable materials where non-destructive disassembly is possible, should be prioritized in green TEG design, even at some cost to moisture barrier performance.

#### Flexible module geometry

In flexible modules subjected to repeated bending, both electrode contact quality and the thermal interface geometry change with deformation. Device-level characterization should include bending cycling data at realistic radii of curvature and performance retention after defined bending cycles should be reported.

Module-level thinking must be integrated into material design from the outset. A material that performs well in isolation but proves incompatible with scalable contact formation, balanced n/p pairing, or durable encapsulation has limited practical value regardless of its intrinsic thermoelectric metrics.

### Long-term operational stability

Long-term operational stability remains one of the most critical underreported barriers for green thermoelectric devices. The primary degradation mechanisms differ across material classes and must be understood to design meaningfully durable systems.

#### Organic and polymer systems

PEDOT:PSS undergoes “dedoping” under ambient humidity, with electrical conductivity and Seebeck coefficient both sensitive to water absorption. Secondary dopants such as DMSO, ethylene glycol that are introduced to enhance performance can evaporate or undergo phase separation over time. Most published stability data for PEDOT:PSS-based TEG films extend to 100–500 h under ambient conditions with very few reports exceeding 1000 h.

#### Ionic systems

Gel-based ionic thermoelectrics face leakage under thermal cycling as the gel matrix expands and contracts, which disrupts the electrode-electrolyte interface and causes progressive electrolyte loss. Thermogalvanic cells based on liquid electrolytes are particularly vulnerable. Reported cycle stability data are sparse, and lifetime under realistic intermittent thermal load conditions has rarely been systematically characterized.

#### Biomass-derived materials

Bio-derived substrates and matrices can undergo hygroscopic swelling, microbial degradation, and oxidation under operational conditions, particularly in non-hermetic device configurations. This is a design feature for end-of-life biodegradability but a liability during operational lifetime.

These reported lifetimes fall short of typical deployment requirements. Consumer electronics applications generally demand more than 5,000 h of reliable operation. Likewise, infrastructure or industrial monitoring applications require substantially longer. Closing this gap requires: (1) encapsulation strategies that exclude moisture without precluding material recovery; (2) crosslinking or composite reinforcement of polymer and gel matrices to suppress mechanical degradation under thermal cycling; and (3) systematic accelerated lifetime testing under realistic humidity, temperature, and mechanical conditions, reported alongside thermoelectric performance data rather than deferred to separate stability studies. The green character of a device that degrades within hundreds of hours is difficult to justify on any life cycle basis.

### Techno-economic feasibility and technology readiness

A realistic assessment of green thermoelectrics must also weigh manufacturing cost and reliability against commercially established Bi_2_Te_3_-based technology. Bi_2_Te_3_ module manufacturing benefits from decades of process maturity, established supply chains, and mature quality control, giving it a persistent cost-per-watt advantage for high-output applications despite the criticality of its constituent elements. Green alternatives are unlikely to compete on a direct cost-per-watt basis in this regime in the near term. However, for the low-power application domains identified throughout this perspective, wearables, distributed sensors, disposable devices, the relevant economic comparison is total system cost rather than cost-per-watt, and here solution-processable green materials such as PEDOT:PSS composites and printed carbon systems offer a genuine manufacturing cost advantage. These routes are compatible with ambient-pressure, roll-to-roll processing that avoids the vacuum deposition and high-temperature sintering steps required for conventional Bi_2_Te_3_ modules, translating into lower capital equipment requirements and potentially lower unit cost at scale, even though per-watt output remains lower. Reliability considerations cut the other way: Bi_2_Te_3_ modules offer well-characterized multi-year operational lifetimes, while the stability limitations discussed in long-term operational stability remain a genuine barrier to commercial reliability claims for green alternatives. Closing this reliability gap, more than closing the performance gap, may be the more decisive factor in realistic near-term competitiveness.

This reliability and maturity gap is reflected in the current technology readiness level (TRL) of each material class. Bio-polymer and organic systems, such as PEDOT:PSS composites, and biomass-derived carbon materials both sit at approximately TRL 3–4, corresponding to laboratory validation; the former is limited primarily by humidity-driven dedoping and a lack of standardized, recyclability-compatible encapsulation, while the latter is limited by batch-to-batch feedstock variability and regionally constrained supply. Recycled inorganic thermoelectrics are somewhat further advanced, at approximately TRL 4–5, with purification cost and consistency at scale, along with secondary waste management, remaining the primary barriers. Ionic thermoelectric systems remain the least mature, at approximately TRL 2–3, constrained by electrolyte leakage under thermal cycling, low power density, and a scarcity of long-term stability data under realistic operating conditions. Across all four classes, the engineering barriers to commercialization are rooted less in intrinsic material performance and more in stability, reproducibility, and interfacial or module-level integration, reinforcing a central argument of this perspective: continued gains in ZT alone are unlikely to be the limiting factor in moving green thermoelectrics toward commercial readiness.

### Outlook

As the field matures, device-level reporting should complement the material-level checklist proposed in green fabrication methods. Transparency must extend beyond intrinsic thermoelectric metrics to include contact resistance, bending stability, humidity resistance, and end-of-life disassembly. Incorporating these parameters alongside standard *ZT* and power factor values will ensure that sustainability claims are supported and that device performance is evaluated under realistic operating conditions.

Future progress in green thermoelectrics will likely depend on integrating several complementary strategies, including: (1) hybrid architectures combining bio-derived matrices with high-mobility conductive fillers; (2) interfacial engineering to reduce contact resistance and stabilize doping; (3) module geometries optimized for low temperature gradients below 50 K; (4) encapsulation approaches that balance environmental durability with end-of-life disassembly; and (5) life cycle centric material selection that treats supply chain, processing burden, and circularity as primary design inputs.

Ultimately, green thermoelectric materials do not need to outperform every conventional inorganic system to become technologically important. Their greatest potential lies in flexible, lightweight, low-temperature, disposable, and distributed energy-harvesting applications, where low toxicity, scalable fabrication, and circular material sourcing may outweigh the pursuit of maximum *ZT* alone. The next stage of development should therefore aim not merely for higher *ZT*, but for better-characterized, more honestly reported, and designed devices that can make a genuine contribution to sustainable energy systems.

Taken together, the device-level challenges discussed in this section i.e., contact resistance, module engineering, and long-term stability collectively illustrate that material-level *ZT* is a necessary but insufficient basis for evaluating green thermoelectric viability. Table 3 consolidates these dimensions into a single comparative reference, summarizing key performance, stability, recyclability, and life cycle indicators across all four green material classes benchmarked against conventional thermoelectric materials. It should be noted that the ZT and power factor values compiled throughout this perspective, including those in Table 3, are drawn from studies using heterogeneous measurement protocols such as differing temperature ranges, characterization methods, sample geometries, and environmental controls, and should therefore be read as representative orders of magnitude rather than protocol-matched benchmarks. The key takeaway is that green materials are neither universally superior nor inferior; rather, they exhibit distinct trade-off profiles that depend on the specific application. Bio-polymer and organic systems offer the lowest processing burden but the shortest demonstrated lifetimes, whereas biomass-derived carbons present strong circularity credentials but modest performance. Recycled inorganic systems recover much of the performance advantage of their virgin counterparts at meaningfully reduced life cycle cost, and ionic systems deliver exceptional Seebeck coefficients in exchange for power density and stability limitations that remain engineering challenges. Bridging the gap between laboratory promise and deployable green thermoelectric devices will require the field to treat these trade-offs explicitly i.e., designing for the full framework rather than optimizing any single dimension in isolation.

## Conclusion

Green thermoelectric research has moved beyond simple material substitution. The field now encompasses water-based processing, bio-assisted synthesis, biomass-derived carbon, recycled inorganic compounds, biodegradable substrates, and ionic thermoelectric systems. These advances are significant, but they do not yet constitute systemic sustainability. A material cannot be considered green solely because one precursor is renewable or because the measured *ZT* is acceptable. Process energy, solvent burden, waste treatment, stability, scalability, and end-of-life recovery must be included in the same design framework. The message is that green thermoelectrics should not be judged against conventional inorganic materials on peak *ZT* alone. Their value lies in specific settings: low-grade heat, flexible, and wearable systems, distributed sensors, disposable or low-cost modules, and devices where low toxicity, low-temperature processing, or circular sourcing matters. Recycled inorganic materials may serve higher-output applications, while organic, bio-derived, and ionic systems may be better suited to soft or low-power devices. If green thermoelectric materials are developed together with process documentation, device-level validation, and circular recovery routes, they can become a credible component of decentralized clean energy harvesting. The field should therefore move from green labels toward accountable design that emphasizes useful power, transparent processing, durable operation, and reduced total environmental impact. A natural and valuable next step for the field is the development of a fully weighted, multi-criteria decision model i.e., supported by sensitivity analysis and validated across a representative sample of material systems, that formalizes the qualitative framework proposed in this perspective into a quantitative decision-support tool. Such a model would require normalized scoring functions for heterogeneous variables including ZT, embodied carbon, material criticality, manufacturing cost, and technology readiness, together with defensible weighting coefficients appropriate to different deployment scenarios. We view this as a substantial research undertaking in its own right, building directly on the reporting checklist, case studies, and comparative data established here, and we encourage the community to pursue it as a distinct and valuable contribution.

## Data and code availability

There is no new data generated in this review.

## Acknowledgments

N.S.S. and I.A.F. thank Universiti Tunku Abdul Rahman for the provision of the Post-doctoral Research Scholarship. This work was supported by JST NEXUS, Japan grant no JPMJNX25F4.

## Author contributions

N.S.S.: conceptualization, writing – original draft preparation. I.A.F.: visualization, writing – reviewing and editing. N.V.T.: writing – original draft preparation, writing – reviewing and editing. T.O.: writing – original draft preparation. M.F.M.S.: writing – reviewing and editing. H.J.: conceptualization, writing – original draft preparation, writing – reviewing and editing, supervision, project administration. All authors reviewed and commented on previous versions of the manuscript. All authors read and approved the final manuscript.

## Declaration of interests

The authors declare no competing interests.

## Declaration of generative AI and AI-assisted technologies in the writing process

During the preparation of this work, the authors used AI tool to improve the language and clarity of the content. After using this tool, the authors reviewed and edited the content as needed and take full responsibility for the content of the publication.
